# The Ameliorating Effect of Plasma Protein from *Tachypleus tridentatus* on Cyclophosphamide-Induced Acute Kidney Injury in Mice

**DOI:** 10.3390/md17040227

**Published:** 2019-04-15

**Authors:** Xinhuang Kang, Mengyao Jing, Guoguang Zhang, Lanzheng He, Pengzhi Hong, Chunmei Deng

**Affiliations:** 1 Department of Applied Chemistry, College of Chemistry and Environment, Guangdong Ocean University, Zhanjiang 524088, China; kang.xinhuang@aliyun.com (X.K.); jmy_0420@163.com (M.J.); zgg929@163.com (G.Z.); helz66@163.com (L.H.); 2 Department of Food Quality and Safety, College of Food Science and Technology, Guangdong Ocean University, Zhanjiang 524088, China; hongpengzhi@126.com

**Keywords:** plasma, cyclophosphamide, kidney, autophagy, apoptosis, *Tachypleus tridentatus*

## Abstract

In the study, the protective effect of plasma protein from *Tachypleus tridentatus* (PPTT) on acute kidney injury (AKI) and the related molecular mechanisms were first investigated by Western blotting analyses, TdT-mediated dUTP Nick-End Labeling (TUNEL) assay, and immunohistochemistry. It was found that PPTT had an obviously inhibitory effect on Reactive oxygen species (ROS) in cyclophosphamide (CTX)-exposed mice. Furthermore, results demonstrated that the renal cell death mode is due to inducing apoptosis and autophagy inhibited by dose-dependent PPTT in mice treated with CTX by decreasing the protein expression of bax, beclin-1, and LC3 and increasing the expression of bcl-2. Moreover, the p38 MAPK and PI3K/Akt signaling pathways were observed to take part in the PPTT-induced renal cell growth effect by enhancing the upregulation of the expression of Akt and p-Akt as well as the downregulation of the expression of p38 and p-p38, which indicated a PPTT ameliorating effect on AKI CTX-induced in mice through p38 MAPK and PI3K/Akt signaling pathways. Briefly, this article preliminarily studies the mechanism of the PPTT ameliorating effect on AKI CTX-induced in mice, which helps to provide a reference for PPTT clinical application in AKI therapy.

## 1. Introduction

The kidney plays an important role in human life activities by excreting metabolic waste, regulating body fluids, and secreting endocrine hormones to maintain the stability of the internal environment and normal metabolism. During metabolism and excretion, once accumulated in the kidney for a long time, toxic metabolites easily cause a sudden increase in urine output, urea nitrogen, and asymptomatic creatinine and further induce acute kidney injury (AKI). In recent years, it is generally believed that the abuse of drugs in clinical practice has resulted in an increasing incidence of AKI, and as high as 20% of these cases result in renal failure. In China, nearly 4 million people suffer from AKI each year, and the number is rising year by year. In particular, the incidence and mortality of AKI has exceeded 60%, and international multicenter studies have warned that AKI is a common acute, dangerous, severe disease that will bring serious medical, social, and economic burdens [1,2].

Cyclophosphamide (CTX) is a broad-spectrum anti-tumor drug widely used in clinical practice [3], but ample evidence suggests that CTX can be further converted to chloroacetaldehyde, which produces nephrotoxicity, neurotoxicity, bladder toxicity, and AKI [4,5,6], which severely influence the treatment options of CTX in clinical practice [7,8]. Therefore, it is urgent to find an effective way to alleviate its adverse effects. In the existing literature, anti-oxidants such as blueberry broccoli [9], thyme [10], and paeoniflorin [11] have been used for the relief of CTX-induced renal damage. However, there is no exact evidence that PPTT can alleviate the kidney toxicity caused by CTX. *Tachypleus tridentatus* is an extremely precious marine animal, and its important economic value may be mainly embodied in blue blood containing a variety of important biologically active substances [12] that are mainly used as a raw material to prepare tachypleus amebocyte lysate, and a large amount of remaining plasma is then discarded, resulting into expensive marine resources wasted. At present, based on no related research at home and abroad about PPTT, this study was designed to adopt PPTT prepared from the discarded plasma to assess its ameliorating effect on AKI in CTX-exposed mice.

In this experiment, a mouse model was established by intraperitoneal injection of CTX. After intervention with PPTT, whether PPTT affects the level of oxidative stress in mouse kidney tissues and the expression of related apoptosis and autophagy proteins was explored in order to find a suitable method for alleviating renal damage in CTX-exposed mice and improving kidney function. We expect to develop health care products and medicine for relieving AKI from the discarded plasma.

## 2. Results

### 2.1. Attenuation of PPTT on the Negative Effects on Body Mass, Kidney Mass, Kidney Index in CTX-Induced Mice

After mice were sacrificed, kidney tissues were removed and weighed to determine kidney index. Results showed that the kidney index in the CP group significantly increased compared with that in the NC group (*p* < 0.01), reflecting the kidney edema, even hyperplasia and congestion, and inducing AKI. However, the kidney index at any detailed dosage of PPTT began to reduce compared with the CP group (*p* < 0.01 and *p* < 0.05), indicating that PPTT could relieve renal damage (Table 1).

### 2.2. PPTT Relieves CTX -Induced Renal Oxidative Stress

CTX, an alkylating agent widely used in anti-tumor clinical can cause a body’s oxidative stress [9], and CTX-induced oxidative stress is the main cause of AKI [5]. Previous studies have shown that CTX-induced tissue damage can bring about the change of malondiadehyde (MDA), catalase (CAT), superoxide dismutase (SOD), and glutathione peroxidase (GSH-Px) in tissues [9,10,11].

Next, we investigated the effect of PPTT on CTX-induced oxidative stress by changes in lipid peroxidation and the activity of antioxidant enzymes GSH-Px, SOD, and CAT. As shown in Table 2, kidneys treated with CTX exhibited a significant increase in the level of the lipid peroxidation product MDA, a commonly known marker of oxidative stress, and reduced the activities of endogenous antioxidant enzymes SOD, CAT, and GSH-Px in kidney tissues (*p* < 0.01, vs. the NC group) as expected. Our observations also suggested that PPTT, isolated and purified from the discarded plasma, may independently suppress MDA induced by CTX and increase GSH-PX, SOD, and CAT activities. Dose-dependent PPTT pre-treatment at 50, 100, and 200 mg/kg for 10 consecutive days ameliorated all of these alterations of renal oxidative stress. These data show that the anti-oxidative activity of PPTT might, at least partly, be responsible for its renal protective effect. PPTT at different doses significantly abolished the CTX-induced oxidative stress to some extent (Table 2).

### 2.3. Western Blotting Analyses

**Figure 1:**
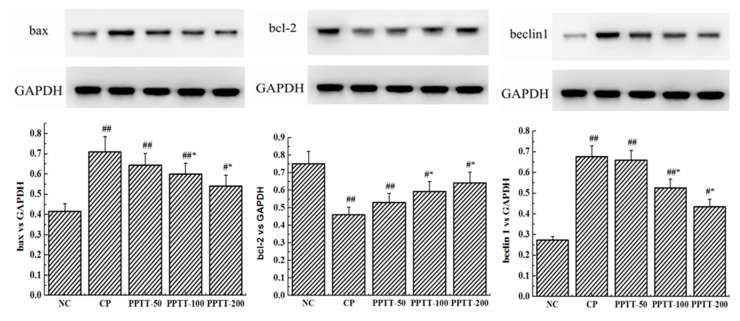

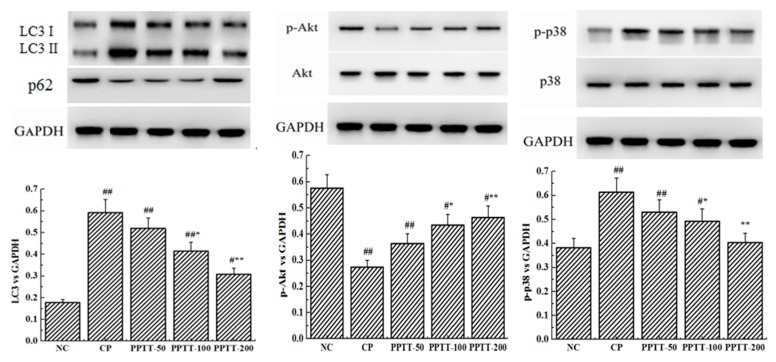
Impairment of PPTT on CTX-induced apoptosis and autophagy in kidneys of mice. Proteins were prepared from kidney tissues to detect expression levels of bax, bcl-2, beclin-1, and LC3 genes and phosphorylation levels of p38 and Akt proteins by Western blotting analysis. Protein levels were all normalized with glyceraldehyde-3-phosphate dehydrogenase protein content. Note: Significant difference is represented with # and *, *p* < 0.05; extremely significant difference with ## and **, *p* < 0.01. In addition, ## and # stand for difference versus normal group; ** and * for difference versus the CP group.

In order to establish whether the ameliorating effect of PPTT on AKI CTX-induced in mice could be validated, a study was conducted to detect the expression levels of two key apoptosis-associated genes (bax and bcl-2) in kidney tissue by Western blotting (Figure 1). Similar to oxidative stress results, the significantly upregulated protein level of pro-apoptotic bax following CTX challenge was obviously decreased, while the downregulated protein level of anti-apoptosis gene bcl-2 was increased by PPTT treatment (*p* < 0.05 or *p* < 0.01, vs. the NC group and the CP group) at different doses.

Consistent with previous reports about kidney cell apoptosis, our results show that mice following CTX treatment revealed a significant increase in autophagy as measured by the WB test when compared to untreated mice in the NC group (Figure 1). These results were confirmed by immunohistochemical analysis showing that the autophagy-associated genes LC3 and Beclin-1 significantly increased in CTX-induced mice compared with the NC group, but those genes were significantly attenuated when PPTT was administered instead of CTX. These data suggest that CTX-induced apoptosis and autophagy in kidney can be effectively inhibited by PPTT.

As shown in Figure 1, expression levels of P38 and phospho-P38 increased, but Akt and phosphor-Akt expression decreased in CTX-treated mice. However, PPTT exposure converted the effects of CTX on the two important proteins. All these results suggested that p38 MAPK and PI3K/Akt signaling pathways in kidney were confirmed to play an important role in modulating apoptosis and autophagy in PPTT-exposed mice injured by CTX.

### 2.4. TUNEL Assay

**Figure 2:**
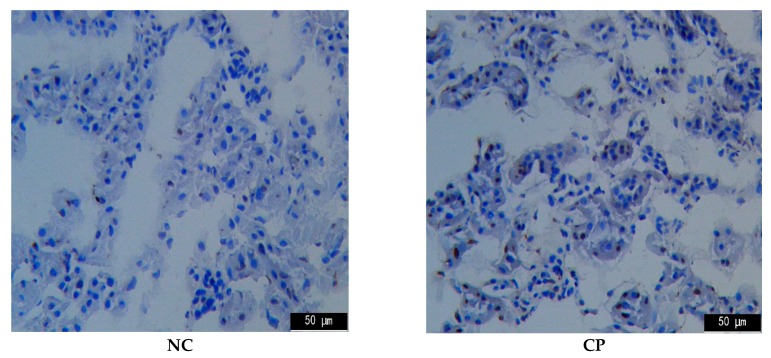

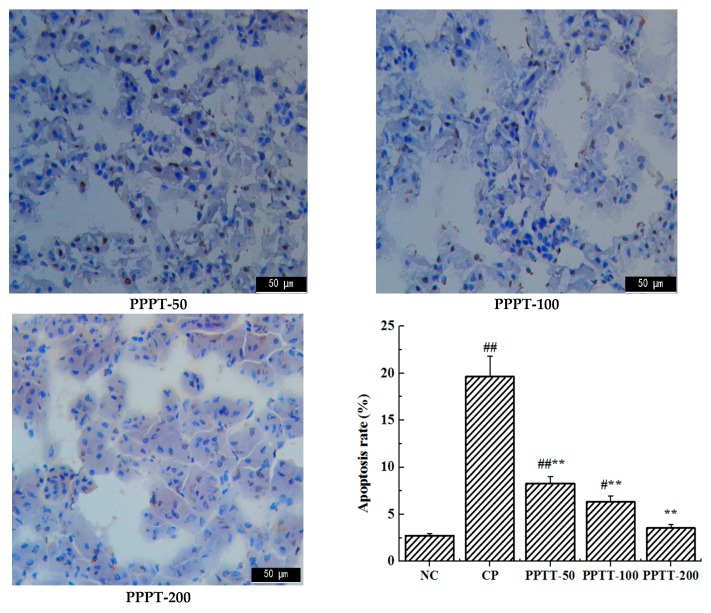
TUNEL analysis for apoptosis in kidney tissues in mice. Note: Significant difference is represented with # and *, *p* < 0.05; extremely significant difference with ## and **, *p* < 0.01. In addition, ## and # stand for difference versus normal group; ** and * for difference versus the CP group.

Apoptosis is a programmed cell death that could be elicited by oxidative stress, inflammation, and other factors, and it is controlled by the balance between the pro- and anti-apoptotic Bcl-2 gene family [13]. As the first cellular response to kidney toxic damage, renal cell apoptosis is one of the key contributing factors for the development of AKI and is also a significant feature in CTX-induced kidney injury. To evaluate the anti-apoptotic effect of PPTT, we determined the gene expression levels of bcl-2 and bax in the kidney of PPTT exposure mice injured with CTX (Figure 2).

The results from TUNEL confirmed that CTX challenge could induce massive renal cell apoptosis. However, treatment with PPTT greatly reduced the number of TUNEL-positive apoptotic renal cells in line with results from Western blotting analyses (*p* < 0.01, vs. the CP group), which indicated that PPTT could prevent apoptosis in CTX-exposure renal tissue and relieve CTX adverse effects, showing positive treatment results in kidney damage induced by CTX.

### 2.5. Immunohistochemistry

The anti-apoptotic gene Bcl-2 in mouse kidney induced by CTX was significantly downregulated (*p* < 0.01), and the abundance of apoptotic gene bax was also significantly increased (*p* < 0.01). Conversely, the effect was markedly reversed in mice treated with PPTT (Figure 1).

The promotion of expression of two autophagy-related genes, LC-3 and Beclin-1, in CTX-administered kidneys suggests that apoptosis also triggers autophagy, possibly even autophagic cell death.

**Figure 3:**
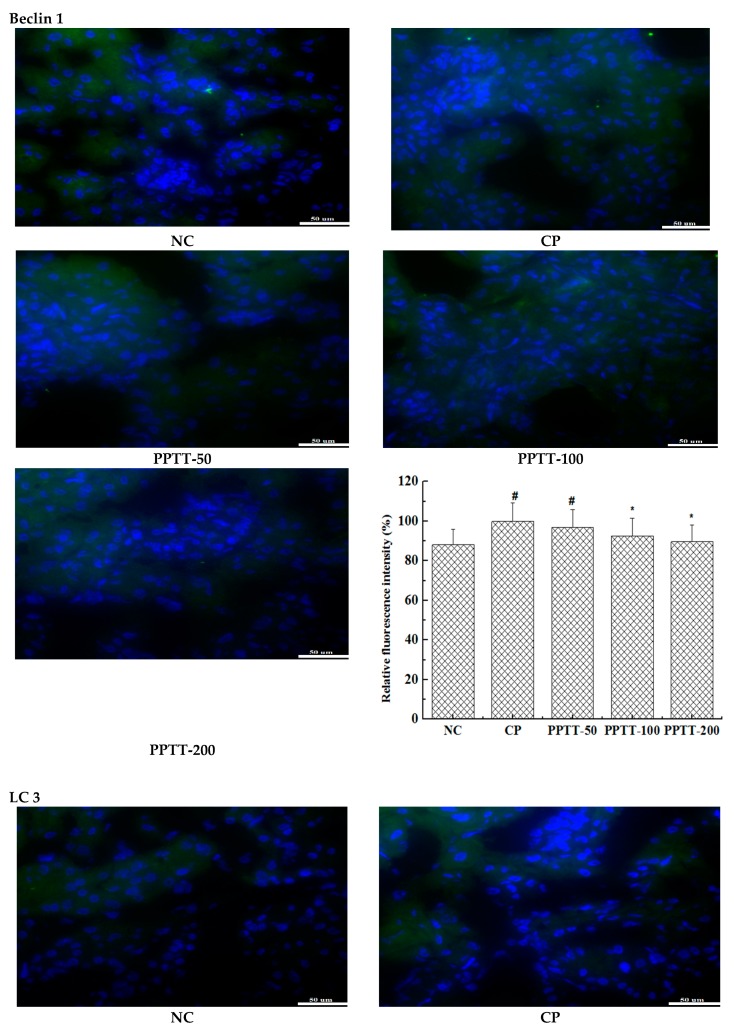

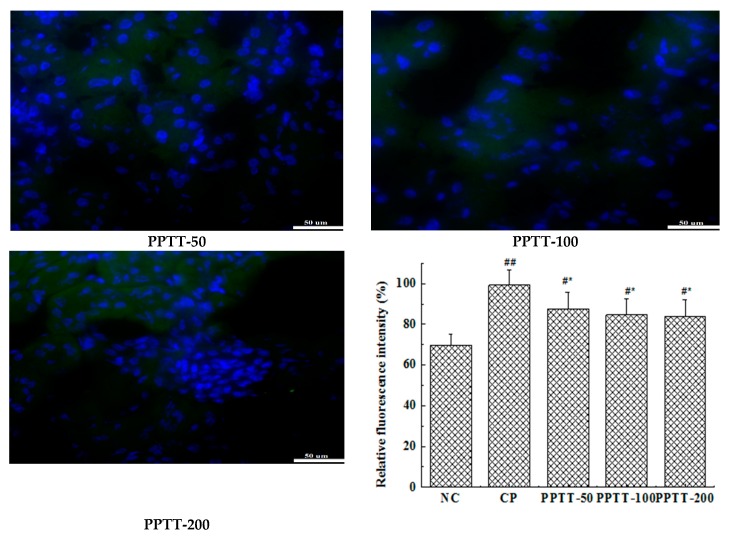
Autophagy of kidney cells CTX mediated was reduced by PPTT in mice. Kidneys from mice exposed to CTX and/or PPTT were subjected to make paraffin sections with routine methods. LC 3 or beclin-1 protein was probed with the corresponding first antibody and the fluorescein labeled secondary antibody and was then observed under an inverted fluorescence microscope, and photos were taken. The green color indicates the antibody and the blue color indicates the nucleus. Note: Significant difference is represented with # and *, *p* < 0.05; extremely significant difference with ## and **, *p* < 0.01. In addition, ## and # stand for difference versus normal group; ** and * for difference versus the CP group.

It was also found that the phosphorylation level of p38 or Akt protein was elevated or reduced by using CTX, respectively, but PPTT exposure converted the effects of CTX on the two important proteins. PPTT has a substantial protective role against apoptosis and autophagy in CTX-induced mice by influencing p38 MAPK and PI3K/Akt signaling pathways in kidneys afflicted with AKI (Figure 2 and Figure 3).

## 3. Discussion

The kidney plays an important role in the metabolic process by which unwanted substances and even harmful waste will be excreted for maintaining normal physiological activities of the human body. In addition, kidneys can also remove toxic substances that cause AKI. If kidneys are not functioning properly, the excretion of harmful substances in the human body is affected such that the accumulation of toxins cannot be immediately removed, which may lead to various diseases and seriously endanger our bodies. At present, prevention and treatment of renal diseases has become the focus of many scholars at home and abroad [14,15].

Various stimulating factors in the body will result in oxidation stress due to an imbalance between antioxidant defense mechanisms and excessive production of reactive oxygen species (ROS). ROS can cause lipid breakdown through lipid peroxidation, and the level of MDA in the final products can reflect the severity of oxidative stress damage in tissue cells [16]. SOD, GSH-Px, and CAT are key antioxidant enzymes in the body and play important roles in the process of MDA elimination [17]. CTX is a medication usually used as chemotherapy, and its metabolites are primarily excreted in the urine. It could be converted to phosphamide mustard and acrolein, which is a kind of highly reactive and unsaturated aldehyde, and this conversion causes nephrotoxicity and strongly injures the kidney tissue [5,6], subsequently resulting in AKI. Compared with the CP group, PPTT can reduce the MDA level and enhance the activity of SOD, CAT, and GSH-Px antioxidant enzymes, thus reducing the oxidative stress and further restoring AKI.

ROS can activate apoptosis and autophagy [18,19,20]. In this work, Western blot was adopted to demonstrate that treatment with CTX resulted in significant bcl-2 content reduction and bax content elevation in kidneys compared with the NC group. However, exposure to PPTT blocked these trends and reverted the expression levels of the two genes. On the other hand, two key genes associated with autophagy, LC-3 and beclin-1, were upregulated but inhibited in PPTT-exposed mice.

PI3K/Akt/mTOR is a critical signaling pathway during autophagy. Our data revealed that LC-3 and beclin-1 proteins were upregulated significantly and Akt was dephosphorylated significantly in CTX-exposure mice, which implied that the regulation of PPTT and/or CTX on kidney was correlated with the PI3K/Akt/mTOR signaling pathway. The phosphorylated Akt can mediate the downstream signaling, which causes a series of biological effects including cell resistance to apoptosis and inhibition of autophagy. Our results show that phosphorylation of Akt protein was inhibited in CTX-treated mice, but expression of LC-3 and becline-1 genes was promoted, which implies that CTX might have promoted autophagy via inhibiting the PI3K-I/Akt/mTOR signaling pathway, which was effectively relieved by PPTT in CTX-stimulated mice. Thus, we speculate that CTX can cause excessive ROS production, leaving renal tissue cells in an oxidative state. If this were true, a large number of antioxidants substances such as SOD, GSH-Px, and CAT would be consumed, the balance between reactive oxygen species and antioxidant system would be destroyed, and the PI3K/Akt/mTOR pathway would be inhibited, leading to autophagy. Under the combined action of oxidative stress imbalance and autophagy defects, abnormal cell apoptosis leads to the occurrence of AKI, but PPTT simultaneously inhibited apoptosis and autophagy induced by CTX.

In our study, p38 and p-p38 expression levels were also found to be significantly increased in CTX-mediated AKI, indicating that the mechanisms of CTX might be involved in the activation of p38 MAPK signaling pathway to induce apoptosis, and the activated p38 MAPK signaling cascade could also induce cells to autophagic death. Both apoptosis and autophagy are important processes for maintaining cell survival and homeostasis, and there is an extremely complex relationship between apoptosis and autophagy. Apoptosis can be inhibited by autophagy [21], and autophagy can also promote apoptosis [22]. Therefore, we can assume that PPTT could relieve AKI by the upregulated PI3K/Akt/mTOR and the downregulated p38 MAPK signaling pathways to inhibit apoptosis and autophagy.

## 4. Materials and Methods

### 4.1. Preparation of Plasma Protein from Tachypleus tridentatus (PPTT)

Raw material in the work was the discarded plasma obtained from remainders of tachypleus amebocyte lysate production. PPTT was prepared according to our patent [23]. The discarded plasma were heated in a constant temperature water bath for 3 h, and then filtered and repeatedly washed with water. After copper removal, the obtained protein was subsequently hydrolyzed by protease and centrifugally separated to collect the supernatant. PPTT was finally obtained after the supernatant freeze-drying.

### 4.2. Animal Models and Treatment Protocol

Kunming mice (35–36 days old, 20 ± 2 g) were obtained from the Guangdong Medical Animal Experimental Center of the Guangdong Province (SPF Grade, Certificate No. SCXK-2013-0002, Guangdong, China). All animals received human care according to the criteria outlined in the Guide for the Care and Use of Laboratory Animals published by the National Institutes of Health, and the experiments were approved by the Animals Care and Use Committee of Guangdong Ocean University.

All mice were housed under standardized conditions in a room on a 12 h light/dark cycle with food and water available ad libitum. Following habituation for 3 days, CTX (dissolved in normal saline) was intraperitoneally injected into mice at a dosage of 80 mg/kg∙BW once every day for three consecutive days. Simultaneously, mice in the NC group were intraperitoneally injected with normal saline at the same dosage. Fifty mice were allocated to five groups including a normal group (NC) as a blank group, a CTX-treated group (CP) as a control group, a low PPTT concentration group (PTT-50), a middle PPTT concentration group (PPTT-100), and a high PPTT concentration group (PPTT-200), with 10 mice per group, half females and half males. Mice were then orally administered with PPTT at a dosage of 50, 100, and 200 mg/kg∙BW, respectively, in the PPTT-50, PPPT-100, and PPPT-200 groups once per day for 10 consecutive days. Accordingly, the NC and CP groups were orally administered with the same volume of normal saline. Ten days after administration, all animals were sacrificed, and the kidney tissues were then isolated and weighted for Western blot, TUNEL, and immunohistochemistry analysis and measurement of oxidative parameters.

### 4.3. Detection of Kidney Index

After the last PPTT treatment, all mice were fasted, but water was available ad libitum for 24 h. Mice were executed by way of the dislocated cervical vertebrae, whose kidneys were collected after clearing away fat around them, cleaning with ice-cold normal saline, then dried with filter paper, and weighed. The kidney index was calculated as follows: kidney wet weight (g)/body mass (g) × l00%.

### 4.4. Measurement of Parameters Related to Oxidative Stress in Liver

Partial renal tissues in pre-cooled isotonic saline were mechanically homogenized. The protein contents in kidney homogenates were measured with routine laboratory methods using the bicinchoninic acid protein assay kit. The supernatant of kidney homogenates was subjected to measure the activities of malondiadehyde (MDA), catalase (CAT), superoxide dismutase (SOD), and glutathione peroxidase (GSH-Px) by an Enzyme-Linked Immunosorbent Assay (ELISA). All ELISA kits were purchased from Nanjing Jiancheng Bioengineering Institute and tested according to the given instructions by DNM-9606 Enzyme Labeling Instrument bought from Wuxi Ruimei Bioelectronics Technology Co., Ltd.( Wuxi, China).

### 4.5. Western Blotting Analysis

The kidney tissues in pre-cooled isotonic saline were homogenized and then centrifugated at 4 °C, 3000 rpm for 15 min. The protein concentration in the obtained supernatants was measured using the BCA assay kit. The proteins were separated by 12% SDS-PAGE and then transferred to a nitrocellulose membrane by electroblotting. After blocking with TBS-T containing 5% non-fat dry milk for 1 h at room temperature, the membrane was incubated with primary antibodies (see Table 3) of cleaved bax, bcl-2, becline 1, LC3, p-p38, and p-Akt for 1 h and then incubated with a secondary antibody for 1 h at room temperature. Finally, the membrane was treated with the reagents in an electrogenerated chemiluminescence (ECL) chemiluminescence detection kit and then scanned. Glyceraldehyde-3-phosphate dehydrogenase (GAPDH) was used as the internal reference.

### 4.6. TUNEL Assay

The apoptosis was assessed by TUNEL assay. The kidney frozen sections were successively fixed, washed, and incubated with proteinase K for 30 min at 37 °C. After incubation in TdT enzyme reaction solution for 60 min at 37 °C in the dark, sections were exposed to a streptavidin-HRP solution for 30 min at 37 °C in the dark followed by staining with DAB solution at room temperature for 10 min. Finally, the apoptotic cells were viewed and counted, and photos were taken under an optical microscope.

### 4.7. Immunofluorescence Assay

The paraffin liver sections were dewaxed, rehydrated, and then fixed with 95% alcohol solution at room temperature for 15 min followed by blocking with 3% H_2_O_2_ at room temperature for 10 min. Following washing three times for 5 min in PBS, the target protein was probed with a monoclonal antibody, incubating overnight at 4 °C, and then captured by the fluorescence-labeled secondary antibody at 37 °C for 30 min. An antifade solution was added into each section, dehydrated, coverslipped, and viewed under a DMIL LED inverted fluorescence microscope. Green staining indicates the antibody, and blue staining indicates the nucleus. Their relative fluorescence intensities were analyzed.

### 4.8. Statistical Analysis

All experimental data were expressed as the mean ± standard deviation. Data were analyzed using t-test of variance (ANOVA) of the SPSS software, version 19.0 (SPSS Inc., Chicago, IL, USA) to compare two different groups of samples. Statistically, differences of *p* < 0.05 were considered significant and differences of *p* < 0.01 were considered extremely significant.

## 5. Conclusions

Studies in the work demonstrated that PPTT, plasma protein from *Tachypleus tridentatus*, has a relieving effect on CTX-induced AKI. Moreover, results reveal that multiple regulatory mechanisms might be involved in the in vivo renoprotective effect of PPTT: (1) anti-oxidation; (2) anti-apoptosis; (3) anti-autophagy. Besides the PI3K/Akt/mTOR signaling pathway, the p38 MAPK signaling pathway took part in the ameliorating effect of PPTT against AKI induced by CTX. Overall, it was disclosed for the first time that PPTT as an AKI relieving substance could provide new insights on renal treatments. Additionally, PPTT could function as an anti-apoptotic and anti-autophagic agent with a renal protective effect by way of influencing the PI3K/Akt/mTOR and p38 MAPK signaling pathways.

## Figures and Tables

**Table 1:** Plasma protein from *Tachypleus tridentatus* (PPTT) effects on kidney index in mice exposed to cyclophosphamide (CTX).

Groups	Body Mass (g)	Kidney Mass (g)	Kidney Index (%)
**NC**	24.71 ± 1.36	0.20 ± 0.01	0.81 ± 0.05
**CP**	20.54 ± 1.04 ##	0.24 ± 0.02 ##	1.17 ± 0.10 ##
**PPTT-50**	20.94 ± 0.83 ##	0.23 ± 0.02 ##	1.10 ± 0.05 ##
**PPTT-100**	21.43 ± 1.19	0.22 ± 0.01 #*	1.03 ± 0.06 #
**PPTT-200**	22.67 ± 2.05	0.21 ± 0.02 **	0.93 ± 0.09 *

Note: Significant difference is represented with # and *, *p* < 0.05; extremely significant difference with ## and **, *p* < 0.01. In addition, ## and # stand for difference versus normal group; ** and * for difference versus the CP group.

**Table 2:** Effect of PPTT on the levels of renal malondiadehyde (MDA), catalase (CAT), superoxide dismutase (SOD), and glutathione peroxidase (GSH-Px) in CTX-treated mice.

Groups	Doses (mg/kg∙BW)	MDA (nmol/mg prot)	GSH-Px (U/mg prot)	SOD (U/mg prot)	CAT (U/mg prot)
NC	/	3.37 ± 0.32	481.46 ± 35.28	901.05 ± 53.27	38.38 ± 2.80
CP	/	4.72 ± 0.71 ##	346.88 ± 32.61 ##	786.68 ± 75.34 ##	22.01 ± 3.15 ##
PPTT-50	50	4.01 ± 0.47 #*	336.70 ± 28.97 ##	836.44 ± 77.59 #	22.69 ± 2.71 ##
PPTT-100	100	3.83 ± 0.44 #	385.57 ± 35.04 ##*	841.13 ± 68.31 #*	23.28 ± 2.25 ##
PPTT-200	200	3.68 ± 0.39 **	406.76 ± 34.08 #*	881.84 ± 84.48 **	27.20 ± 2.32 ##*

Note: Significant difference is represented with # and *, *p* < 0.05; extremely significant difference with ## and **, *p* < 0.01. In addition, ## and # stand for difference versus normal group; ** and * for difference versus the CP group. The symbol / denotes that the animals in this group were not treated with PPTT.

**Table 3:** Antibody information.

Antibody Name	Antibody Species	Brand	Item No.
bax	rabbit	Bio swamp	PAB30040
bcl-2	rabbit	Bio swamp	PAB30041
Beclin 1	rabbit	Bio swamp	PAB35215
LC3	rabbit	Bio swamp	MAB37400
P62	rabbit	Bio swamp	PAB33349
Akt	rabbit	Bio swamp	MAB37305
p-Akt	rabbit	Bio swamp	PAB43181-p
P38	rabbit	Bio swamp	MAB37199
p-p38	rabbit	Bio swamp	PAB43506-p
GAPDH	rabbit	Bio swamp	PAB36264
Goat Anti-Rabbit IgG	goat	Bio swamp	PAB160011
